# Understanding how outcomes are measured in workplace physical activity interventions: a scoping review

**DOI:** 10.1186/s12889-018-5980-x

**Published:** 2018-08-25

**Authors:** Stacey Johnson, Jean-Philippe Regnaux, Adrien Marck, Geoffroy Berthelot, Joana Ungureanu, Jean-François Toussaint

**Affiliations:** 1 Institut de Recherche bio-Médicale et d’Épidémiologie du Sport (IRMES), Institut National du Sport de l’Expertise et de la Performance (INSEP), 11 avenue du Tremblay, 75012 Paris, France; 2 Université Paris Descartes, EA 7329, Sorbonne Paris Cité, Paris, France; 3 École des hautes études en santé publique (EHESP), Université Sorbonne Paris Cité, Rennes, France; 4 Centre de Recherche Épidémiologie et Statistique Sorbonne Paris Cité (CRESS), METHODS Team, INSERM U1153, Université Sorbonne Paris Cité, Paris, France; 5 Centre d’Investigations en Médecine du Sport (CIMS), Hôtel-Dieu, Assistance Publique - Hôpitaux de Paris, Paris, France; 6 Research LAboratory for Interdisciplinary Studies (RELAIS), Paris, France

**Keywords:** Workplace physical activity program/promotion, Workplace health promotion, Outcome measures, Evaluation tools, Heterogeneity, Sustainability, Scoping review

## Abstract

**Background:**

An inverse relationship exists between physical activity and many non-communicable diseases, such as obesity. Given the daily time spent, a logical domain to reach an adult population for intervention is within and around the workplace. Many government bodies, including the World Health Organization (WHO), include worksite health promotions (WHPs) targeted at increasing physical activity as a public health intervention. The aim of this scoping review was to determine what was measured (outcomes) and how they were measured (evaluation tools) during workplace physical activity interventions in order to identify gaps and implications for policies and practice.

**Methods:**

A scoping review was executed in April 2017 via PubMed, SPORTDiscus, EBSCOhost and the Cochrane Library. This search included articles published between January 2008 to February 2017 in order to coincide with the WHO’s Global Plan of Action on Worker’s Health. Extracted information was arranged into data collection grids. Cross-analysis of measured outcomes with their corresponding evaluation tools was completed. A quality assessment based on study design was executed.

**Results:**

Identification of 732 records was made and ultimately 20 studies and reviews that met criteria were selected. Researchers themed 9 primary measured outcomes. Studies utilized various forms of both objective and subjective evaluation methods. Three primary evaluation methods were categorized: biologic, electronic and declarative tools. The researchers discovered 92 unique tools: 27 objective and 65 subjective, within these parameters.

**Conclusion:**

Study quality, measurement tools and data collection were heterogeneous making analysis of effect comparisons problematic and unreliable. Much of the published research does not employ robust statistical analysis making effects difficult to ascertain. Considering the variety of both measured outcomes and evaluation tools, only educated inferences can be made as to the effectiveness and efficiency of WHPs. More standardized measurement practices are therefore suggested for assessment efficiency.

**Electronic supplementary material:**

The online version of this article (10.1186/s12889-018-5980-x) contains supplementary material, which is available to authorized users.

## Background

The obesity epidemic has reached an all-time high. According to body mass index, just under 2 billion adults are reportedly overweight; among them, half a billion people are obese [1, 2]. Multiple factors are implicated as causes including an increased consumption of calorie dense foods, lower amounts of daily physical activity, increased screen-time both at work and during leisure time, unsupportive environments such as lack of sidewalks or unsafe bike trails as well as genetics [3, 4]. Strong evidence points to physical inactivity increasing the risk of many major non-communicable diseases (NCDs) [1, 5, 6], which make up approximately 70% of the present total burden of disease [7]. Worldwide, NCDs represent a major burden to national healthcare systems. In 2011, the World Economic Forum reported that 63% of deaths could be attributed to NCDs and cost over 30 trillion US dollars [5]. Increased physical activity (PA) can help mitigate many of these disease risk factors and should be a fundamental component of public health work [7].

Much of the adult population falls short of the PA recommendations. Encouraging people to be physically active has numerous benefits beyond health, to include the economy and environment. Previous studies suggest that promoting active modes of transportation can be associated with positive health impact assessments and has the potential to save billions per year by reducing pollution and oil consumption [8, 9]. To improve health through prevention and chronic disease management, adult guidelines recommend 150 min of moderate intensity aerobic physical activity or 75 min of vigorous intensity physical activity (MVPA) per week. Additional benefits are possible with increased duration and strength training [4, 10]. Although such recommendations are widely recognized, adults continue to widen the gap by increasing sedentary behaviors. Latest factsheets from the World Health Organization (WHO) European Region report the percentage of adults attaining appropriate amounts of physical activity fall below acceptable levels for both sexes: Italy 36%, Germany 39%, France 45% and England which fares better at 60% [2, 4, 11].

### Workplace health promotions

Importance has been placed on health promotion since the 1950’s and has undergone multiple charters and declarations. The most notable being the 1986 Ottawa Charter for Health Promotion and the most recent; WHO’s Global Plan of Action on Worker’s Health 2008–2017 [12, 13]. To address the escalation of sedentary behavior, WHO Europe recognized the advantages of the workplace as a medium to reach much of the adult population at multiple levels both directly and indirectly to influence behaviors [4]. Workplace Health Promotion programs (WHPs) are employer initiatives directed at improving the health and wellbeing of workers and their dependents. At their core, they support primary, secondary and tertiary prevention efforts [14].

WHPs often focus on factors such as physical activity, sports, exercise classes and behavior change to increase physical activity levels [15–17]. The work setting is also advantageous for corporations: programs including a physical activity component have the potential to increase employee productivity, reduce absenteeism, act as recruitment and retention tools, reduce health care costs and increase physical activity levels outside the workplace [8, 18, 19]. These settings can also counter common barriers such as lack of time, family duties and low motivation [20, 21].

Many studies on WHPs have been conducted with a myriad of outcomes being measured. In order to determine the impact and long term effectiveness, the link relating the measured outcomes to the promotion or program is very important. Thus far, there is no known standard for evaluating the impact of these promotions/programs or their outcomes. A variety of tools and measures have been used and adapted for specific study purposes, including blood tests, accelerometers and questionnaires, mixing both objective and subjective evaluation tools. Cost, time and assessment for some of these can be of concern for large scale studies due to restricted budgets. Expensive tools can prove to be a barrier for workplaces considering return on investment (ROI) [14, 20, 22, 23]. A vast majority of interventions use self-report measures for levels of PA. Some of these include various forms of diaries, interviews, online journals, pedometer readings and questionnaires. Though possibly biased, the latter are inexpensive, easy to administer, understandable, cost effective and often a preferred method to measure multiple outcomes. Self-report measures have a tendency toward bias and usually overestimate levels of participation compared to objective estimates [24]. Furthermore, universities, research labs and workplaces often create their own questionnaires in order to measure specific outcomes based on personal priority.

Multiple reviews have been published regarding WHPs, yet, none have been expressly concerned with outcomes and evaluation tools. The primary aim of this research was to assess the literature reporting PA changes during WHPs in order to: (1) describe the reported outcome measures, (2) determine the evaluation tools used to evaluate the documented outcomes and (3) detect effects and cross-comparisons of PA interventions. Many public health organizations are concerned with determining which interventions and health promotion programs are most appropriate to achieve sustainable outcomes for both employers and employees alike. This research also notes the secondary outcomes that were reported during WHPs (e.g. improved work productivity, sleep quality, nutritional practices, etc.). Many of these outcomes were considered as a secondary outcome of the increased physical activity.

## Methods

### Eligibility criteria

The methodology of this scoping review was based on methodology set up by The Joanna Briggs Institute [25]. Results have been reported using the PRISMA guidelines for systematic reviews [26]. Prospero has registration for systematic reviews and meta-analysis but currently, none exist for scoping reviews. Three primary criteria for research included: (1) health promotion programs implemented in the workplace, (2) working adults between 18 and 64 years old and (3) physical activity as the primary intervention. Notably, many interventions crossed over into leisure time including, measurements of daily step counts using accelerometers or pedometers. Many studies reported secondary outcomes that were taken into account.

### Search strategy

The search was executed in the English language. Databases included: PubMed, SPORTDiscus, EBSCOhost and the Cochrane Library based on specific keywords. The search included the following word combinations: workplace or worksite or corporation AND physical activity or exercise or fitness or sport AND intervention or program or programme or promotion AND outcomes or benefits or effects AND evaluation methods or evaluation tools or evaluation. Research was conducted on studies and systematic reviews published between January 2008– February 2017. Dates were chosen based on the WHO’s Global Plan of Action on Worker’s Health 2008–2017 which is a call for public health action to include healthy workplace programmes [27].

### Study selection

Two researchers first examined titles then, the abstracts in coordination with each other. Two alternate researchers were advised in cases of dispute. Finally, full text articles were read and screened for eligibility by one researcher with two other researchers offering recommendations on final selection of studies. Consideration was taken to limit study duplication within systematic reviews (12%). A relevant sample was purposively selected from the applicable primary studies and systematic reviews. To determine this chosen sample, both small and large scale studies were included in order to capture a variety of interventions, outcomes and measurement tools. Interventions at both the individual level (self-reported steps) and organizational level (team sports) were considered. Only published studies with reported effects were accepted. The primary target was employed, apparently healthy adults. Studies focusing on particular health issues, such as reducing low back pain or chronically ill populations, were eliminated. Workplace health promotions primarily focusing on preventions such as smoking cessation, immunization, alcohol moderation, nutrition and other lifestyle modifications were not included. Individual studies and systematic reviews that did not include clear outcomes or descriptions of the evaluation tools used were discarded. Based on the data extraction forms, studies with many missing data points were excluded. This included type of measurement tool used, outcome being measured (e.g. number of steps, increase of physical activity, weight lost, etc.), sample size, type of physical activity included in the promotion/program, the type of study (e.g. random control trial, before/after, case control, etc.), length of promotion/program, follow-up measurements and results. Low quality studies such as protocols only, expert opinion or lack of specific study design were also omitted.

### Data extraction

Data extraction forms were created for both primary studies and systematic reviews. Outcome measures were determined for the chosen sample of studies and then coded into structured categories. Similar outcomes appeared with multiple terminologies therefore, based on knowledge, ‘themed’ categories were created. For example, any outcome whose primary purpose was to measure changes in physical activity was included in the ‘Physical Activity Level’ category. When a study reported measuring a particular outcome, a check was given in the coordinating category indicating this particular outcome had been evaluated in the study. While no statistics were used, a qualitative thematic analysis was completed on the outcomes measured and the evaluation tools used for their measurement purposes. Each tool was qualified as objective or subjective and either validated or of unknown validation. Upon completion, scientific validation of the measurement tools was determined against currently accepted best practices such as the International Physical Activity Questionnaire (IPAQ) which is widely used across multiple worldwide organizations (WHO, NIH and CDC). In order to understand implementation and determine areas of potential improvement, coding also included: (1) intervention type, (2) the method of information communication to participants (e.g. email, meetings, signage, etc.), (3) intervention location (4) timeframe of the intervention, (6) sample size and (5) follow-up timeframes including participant attrition. A hypothesis has been drawn to link the outcomes specifically to the physical activity/fitness component of the WHP via the evaluation tool utilized.

The following data were extracted using pre-tested extraction forms:

1. Individual study characteristics (quality grade based on study design, country, worksite, study size and publication journal).
2. Systematic review characteristics (quality grades based on study design, worksites, number of studies included and publication journal).
3. Program characteristics (physical activity promotion program length, location, drop-out rates, follow-up measurements, cost, communication methods, reporting methods and number of simultaneous programs).
4. Primary measured outcomes (physical activity level or fitness). Secondary measured outcomes (motivation/involvement/self-efficacy, self-esteem, nutrition, management/supervisor support of health promotion, anthropometric, bio-chemical parameters, work-related, health-related quality of life and environment).
5. Evaluation tools (questionnaires, electronic devices and biological markers) in accordance with the outcome measured.
6. Synthesis of effects on each of the measured outcomes by reported statistical significance**.**

### Data synthesis

Data were then summarized using descriptive statistics to outline the characteristics of the included studies. This included information on the type of physical activity or fitness measured (e.g. number of daily steps, sitting time, use of active transportation (walking/biking), moderate to vigorous physical activity, etc.), the evaluation tool(s) used for measurement purposes, whether the tool was an objective or subjective method and the validation confirmation of the tool and finally the unit of measure for physical activity. A narrative synthesis was performed to describe the reported outcome measures and the evaluation tools that were used. These were recorded as electronic, biologic or declarative for each of the themed outcomes.

### Study quality

In order to rate the strength of the scientific evidence and the quality of research [25], an objective simple system of graduation was used for the individual studies proposed by Petrisor [28]. This system of grading awards randomized controlled trial (RCT) designs, including individual RCT and cluster RCTs, with an ‘A’, quasi-randomized trials, prospective case control and non-RCT receive a ‘B’, observational studies, controlled before-after, qualitative exploratory, cross-sectional and quasi-experimental designs receive a ‘C’ and other evidence including expert opinion receive a ‘D’. A similar but slightly modified grading system was followed for the systematic reviews where specific study design was reported. If no differentiation was reported, the space has been left blank as no grade can be assigned. Grading can be viewed in the results section.

## Results

### Selection process

**Fig. 1:**
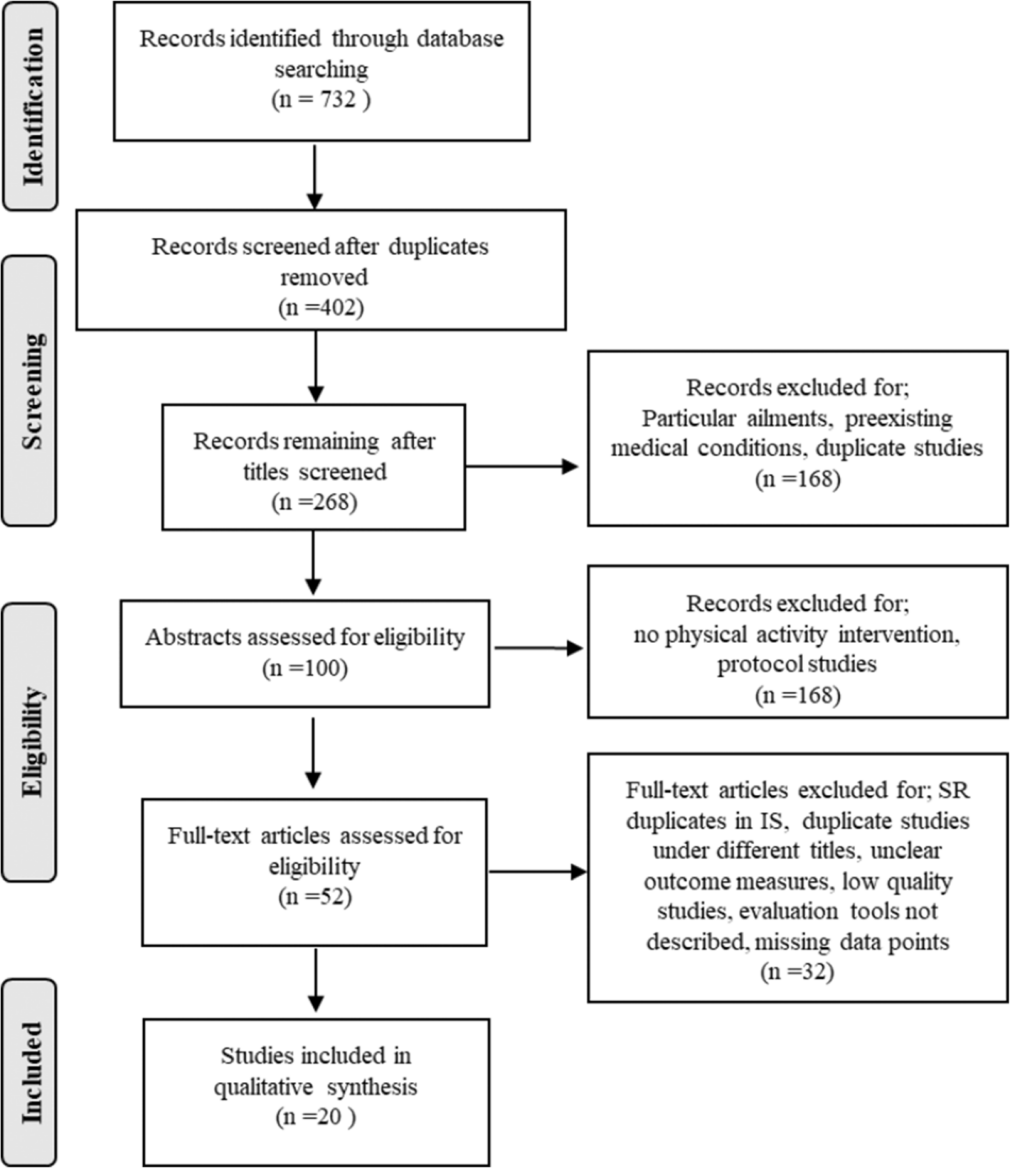
Flow chart displaying study selection process

Search results yielded 204 full text articles in PubMed, 82 results from SPORTDiscus, 416 results through EBSCOhost and 30 results from 9867 records in the Cochrane Library. After removal of duplicates, 402 records remained. Out of these, 268 titles were screened for relevant studies, 100 abstracts were then scanned for acceptable inclusion criteria. Methods were reviewed to ensure characteristics of studies fell within the parameters. Finally, 52 full text articles were read to draw a good representative sample. A sample of 12 individual studies and 8 systematic reviews that fit the scoping review criteria were ultimately chosen. Several studies were excluded due to author using the same study to write several papers, lack of reported evaluation tools, or, in the case of systematic reviews, use of the same individual studies [29] where multiple duplications in the review are used in the individual studies selected by the researchers and no new information was gained. Figure 1 displays the flow chart as to the database search and final article selection.

### Study characteristics

#### Individual studies

Twelve individual studies published between 2008 to 2017 were chosen, originating from: 3 Australia/Oceania, 3 Asia, 4 Europe, 1 North America and 1 South Africa. The workplace settings were: 4 healthcare, 1 education, 2 manufacturing, 2 governmental organizations, 2 retail, 1 airline and 2 studies did not report specific worksites but mentioned white-collar office employees as the target population. Interventions varied widely but all included increasing physical activity as a primary component. Physical activity (PA) modes were: 7 exercise programs, 1 weight-loss program, 1 wellness program, 1 PA counseling, 1 general health promotion program and 1 focused on reduction of sitting time.

The mean sample size of the 12 studies was 408 participants with ranges between 70 [30] to 1442 [31] participants. A sample size of < 200 was observed in 33% (*n* = 4) of the studies. Attrition was reported in all studies and ranged between 4.7–57% with an average of 30.8% over the entire length including follow-up periods which varied between 3 weeks to 12 months post intervention. Study designs included: 5 RCT, 5 observational, 1 case-control and 1 quasi-experimental design (intervention group recruited separately from reference group). Quality grading was such: 5 received an ‘A’, 2 received a ‘B’ and 5 received a ‘C’. Table 1 displays study characteristics for individual studies.

**Table 1:** Individual Study Characteristics

Author	Publication Date	Country	Work Setting	Sample Size	^a^Quality	Intervention
Individual Studies						
Davey, J., et al.	2009	New Zealand	University Employees	134	C	Physical activity-steps
Dishman, R., et al.	2009	United States/Canada	Home Improvement Stores	1442	A	Physical activitysteps/**MVPA
Maruyama, C., et al.	2010	Japan	Health Insurance Association	101	A	Nutrition/Physical Activitysteps
McEachan, R., et al.	2011	UK	Bus Company; Hospital; Local Government; National Government; University	1274	A	Physical activity-MVPA
Morgan, PJ, et al.	2012	Australia	Tomago Aluminum	110	B	Weight lossnutrition/pedometer
Edries, N., et al.	2013	South Africa	3 Clothing Manufacturing Companies	90	A	Wellness program with ***CBT and exercise classes
Chae, D., et al.	2015	South Korea	Airline Company	70	C	Physical activity-steps
Brakenridge, C.L., et al.	2016	Australia	Lendlease-International Property and Infrastructure Group	153	A	Physical activity-reduced sitting time
Hendricksen, I., et al.	2016	Netherlands	Dutch Insurance Company	433	C	Lifestyle-MVPA/sedentary behavior
Hori, H., et al.	2016	Japan	Not listed	490	C	Physical activity-steps
Aittasalo, M., et al.	2017	Finland	12 small-medium size workplaces	396	C	Physical Activity-reduce sitting time
Arrogi, A., et al.	2017	Belgium	Pharmaceutical Company	300	B	Physical activity cousellingswim, bike, run, walk

^a^Grading system is based on study design; A - RCT / B - Quazi-randomized trials / C - Observational Study / D - Other Evidence [28]

**MVPA - Moderate to vigorous physical activity

***CBT - Cognitive behavior therapy

#### Systematic reviews

Eight systematic reviews published between 2008 to 2017 were selected. Reviews originated from various countries; 2 studies did not disclose locations, 4 from Organization for Economic Co-operation and Development (OECD) developed countries, 1 included only European studies and 1 used studies from the USA/Australia/Europe. The worksite settings also varied from education, healthcare, commercial, governmental employees, blue collar settings to manufacturing. The physical activity modes included: increasing steps/walking promotions, active commuting (e.g. biking or walking to and from work), team sports, active workstations and various exercise classes with different intensity levels. The number of studies included in each review ranged from 15 to 138. Studies varied in population sizes with a mean of 5984 participants. The smallest reported study included in a systematic review had 10 participants [32] and the largest study had 79,070 participants [33]. Attrition data was reported in 25% (*n* = 2) of the systematic reviews [34, 35] with an average of 23.5% loss to follow up. Three of the 8 (37.5%) reviews reported study length ranging between 15 days to 12 years [33, 34, 36]. Systematic reviews included all study designs: 7 included RCTs, 5 included observational, 3 controlled before-after, 3 cross-sectional, 1 cohort trial, 1 quasi-experimental and 1 qualitative exploratory. For consistency in this scoping review, grading scales were converted to the ‘A-D’ quality assessment based on the type of study designs reported in each systematic review, this was done in the same fashion as the individual studies. Table 2 displays study characteristics for systematic reviews.

**Table 2:** Systematic Review Characteristics

Author	Publication Date	Quality	Country	Work setting	# of studies	Sample ranges	Intervention
Systematic Reviews							
Conn, V., et al.	2009	Not reported^a^	Not reported	Education/Health Government Manufacturing	138	12–5038	Physical Activity-Fitness Motivation Education
Vuillemin, A., et al.	2011	19 = A 3 = B 11 = C	Europe	Variety	33	14–26,806	Physical Activity
Schroer, S., et al.	2013	9 = A 6 = C	USA/Australia/Europe	Variety	15	10–48,835	Physical Activity/Nutrition
Amlani, N., et al.	2014	9 = A 20 = B 8 = C	Developed Countries	Variety	37	43–79,070	Physical Activity
Malik, S., et al.	2014	31 = A 21 = B 6 = C	Not reported	Education/Health/Government/Commercial	58	32–798	Physical Activity/ Exercise/Counselling/Support Health Promo Message
Brinkley, A., et al.	2016	4 = A 2 = B 12 = C	Developed Countries	Education/Health/Factory/Corporation	18	30–2118	Physical Activity-team sports, group fitness, team walking
Shrestha, N., et al.	2016	14 = A 2 = B 4 = C	High Income Nations	Office workers	20	25–443	Physical Activity- workplace changes policy/counselling/information/Multiple interventions
Reed, J.L., et al.	2017	17 = A 4 = B 3 = C	High Income OECD Nations	Variety	24	26–650	Physical Activity-MVPA/METs

^a^Grading system is based on study design; A - RCT / B - Quazi-randomized trials, prospective case control, non-RCT/ C - Observational, controlled before-after, qualitative exploratory, cross-sectional, quasi-experimental / D - Other Evidence [28]

*NR* authors reported RCT, observational and controlled before-after designs but not specific numbers of each

*MVPA* Moderate to vigorous physical activity, *METs* metabolic equavalents

### Outcomes

#### Measured outcomes

A total of 9 ‘themed’ outcome measures were found across the 20 studies. Physical Activity Level was the primary consideration therefore, this category included; step counts, decreased sedentary activity, increase of moderate to vigorous physical activity (MVPA), metabolic equivalents (METs), aerobic activity level, time spent playing sporting activities and active commuting. The other 8 ‘themes’ included: Fitness, Motivation/Involvement/Self-efficacy, Nutrition, Management Promotion Support, Anthropometric Measurements, Bio-chemical Parameters, Work-related and Health-related Quality of Life (QoL)/Well-being. Fitness was separated out from Physical Activity because it was only found in the systematic reviews and had specific biological tests performed for changes such as VO_2_ max, flexibility, endurance and strength tests such as 1-Repetition Max. As the main inclusion criterion was changes in physical activity level, this was used and measured in 100% of the studies (*n* = 20). Changes in anthropometric measurements such as BMI and body fat percentage were used in 65% (*n* = 13) of the studies. Health Related QoL was measured in 60% of the studies (*n* = 12). Work related outcomes such as absenteeism and work stress were reported in 45% of the studies (*n* = 9). Management involvement was reported in only 20% (*n* = 4) and nutrition in 15% (*n* = 3) of the studies. Changes in PA level was also associated as a causal factor for other outcome measures such as sleep quality [22, 37, 38] or lower absenteeism rates [33, 38–42]. For example, an increase in daily steps leads to a decrease in reported employee sick days. All measured outcomes can be seen in Table 3. Measured Outcomes with a check next to the respective studies.

**Table 3:** Measured Outcomes

Study	PA Level	Mot/Involve/Self-eff	Nutrition	Management	Anthropometric	Biochemical	Work Related	Health Related QoL/Well-Being	Fitness
Individual Studies									
Davey, et al	√	√						√	
Dishman, et al	√	√		√					
Maruyama, et al	√		√		√	√			
McEachan, et al	√				√	√		√	
Morgan, et al	√				√	√	√	√	
Edries, et al	√				√		√	√	
Chae, et al	√	√			√				
Brakenridge, et al	√				√	√	√	√	
Hendricksen, et al	√		√	√	√	√	√	√	
Hori, et al	√							√	
Aittasalo, et al	√						√	√	
Arrogi, et al	√	√		√	√	√		√	
Reviews									
Conn, et al	√				√	√	√	√	√
Vuillemin, et al	√				√				√
Schroer, et al	√		√	√	√				
Amlani, et al	√						√		
Malik, et al	√								
Brinkley, et al	√	√			√	√	√	√	√
Shrestha, et al	√						√	√	
Reed, et al	√				√	√			

*Mot/Involve/Self-eff* Motivation/Involvement/Self-Efficacy, *QoL* Quality of Life

#### Evaluation tools

Three different categories of evaluation tools were found to measure the different outcomes: biologic, electronic and declarative. Electronic devices included anything that measured specific step counts or activity levels such as pedometers and accelerometers, biologic tools included any method that measured levels of bio-chemical or anthropometric factors such as blood-glucose or body fat percentage (BF%) and declarative included all participant reports such as perceived exertion in METs or self-reported questionnaires such as the International Physical Activity Questionnaire (IPAQ) or diaries. They were subsequently categorized into either subjective or objective formats. Researchers discovered 92 unique evaluation tools, of these, 27 were objective and 65 were subjective tools. Table 4 displays the number of *unique* subjective and/or objective tools used for the respective outcomes.

**Table 4:** Number of *unique* objective and subjective evaluation tools for each outcome

Outcome	Objective Tools (*n* = 27)	Subjective Tools (*n* = 65)
Physical Activity Level	6/22%	20/31%
Motivation/Involvement/Self-efficacy		9/14%
Fitness	7/26%	
Nutrition		5/8%
Management		2/3%
Anthropometric	4/15%	
Bio-chemical Parameters	7/26%	
Work-Related	3/11%	12/19%
Health Related QoL/Well-being		17/26%

All biologic and electronic tools were categorized into objective evaluation tools as were 3 of the work related tools; making up 29.3% (*n* = 27). These included connected objects such as pedometers, accelerometers and activity trackers. Biologic testing was also categorized in objective tools which included biomedical measurements such as BMI, BF%, VO_2_ max, cholesterol and blood glucose. Work related measures included in this category were based on human resource data and medical records. Subjective methods made up 71% of the evaluation tools (*n* = 65). Subjective surveys, diaries and questionnaires were the favored tool and used in 100% of the studies (*n* = 20). Physical activity was the primary outcome measure; it was measured via 6 objective and 20 subjective tools. The variety of unique questionnaires, electronic and biologic tools can be seen in (Additional file 1). The specific physical activity outcome for individual studies can be seen in more detail (see Additional file 2) and the same for systematic reviews (see Additional file 3).

## Discussion

Several systematic reviews exist on workplace physical activity interventions. This scoping review is the first of its kind to synthesize information from both systematic reviews and individual studies with the key goal in mind to identify what outcomes are considered as physical activity and how these outcomes are measured. Many previous reviews mention the heterogeneity of WHPs [17, 32, 34–36, 39]. The present review took a broad and inclusive approach and in doing so, several key gaps were identified: (1) difficulty in the comparability of studies, (2) high attrition rates and (3) lack of proven sustainability. Considering the increased interest in this subject for all stakeholders including public health professionals, researchers, corporations and employees, it is necessary to create focused, well-designed studies with adequate post-intervention assessment periods in order to confirm efficacy and sustainability of the increased physical activity results achieved by the interventions [32, 33, 35, 39].

### Study comparability

#### Measured outcomes/evaluation tools

Accurate and reliable measurements of physical activity are important in evaluating programs. Currently, no consensus exists as to the proper way to measure desired outcomes nor which evaluation tools are best used during workplace physical activity promotions. In order to justify time and associated costs [39] for public policy, consistency, reliability and validity is necessary to draw conclusions about the efficacy of interventions. The current lack of homogeneity in both measurement tools as well as what constitutes increased physical activity (steps, MVPA, METs, weight lost, reported time spent playing sports) makes comparisons and effects difficult to interpret which leads to an incomplete ability to measure program impacts [33, 35, 39]. Over the 20 selected studies, 9 themed outcomes were measured through 92 evaluation tools as can be viewed in Additional file 1. A prime example: 26 different evaluation tools were utilized to measure physical activity outcomes. Based on this research, due to cost effectiveness and ease of distribution, self-report tools were the preferred method for measurement of all outcomes, of these, 9 had unknown validation status and 6 were not validated measurement tools. These can be subject to recall bias and overestimations and therefore effects should be interpreted with caution [43, 44]. The IPAQ is validated and currently used in many studies (*n* = 6), but has shown biased estimates of energy expenditure. It was suggested that developing a correction factor when using the IPAQ-LF could alleviate this bias [45]. Because of the reputable nature, validation status and ease of use, the IPAQ could form the basis for studies using questionnaires. In addition, the ability to modify the questionnaire according to the promotion, country context, study design, workplace size and budget while maintaining validation would grant more standardization and allow the discovery of potential new information. This would increase consistency among subjective evaluations and the ability to compare promotions, workplaces and regions.

Objective tools were also found to be used in many programs. Of these, the most widely adopted and validated tool was a pedometer, 12 studies reported using these. They are simple, easy to report data and cost effective. As a starting point, it is suggested that pedometers be incorporated into WHPs in order to give an objective measurement tool easing the ability to compare programs. By focusing on incorporating the more widely used subjective and objective measurement tools, they could form the basis for future research that is more consistent and homogeneous.

As has been demonstrated, the sheer amount of subjective tools and outcome variables makes it nearly impossible to compare interventions. In addition to the multiple subjective tools used for evaluation purposes, concrete markers of physical activity (MVPA) and an objective biomarker (BMI) are strongly encouraged [17, 33–36, 39]. These types of objective measurements will assist in showing outcomes are consistent with increasing physical activity levels that coincide with current health organization recommendations.

#### Study characteristics

As similar systematic reviews found, various study designs were utilized. These often presented inadequate sample sizes, lack of proper control group and no blinding [33]; all play major roles in minimizing bias which are important considerations when designing studies for public policy research [32, 33, 35, 36, 39]. Smaller sample sizes can deliver less accurate results and increases the possibility that impacts will not be transferable to the population [8, 30]. In this research, 1/3 of the individual studies have less than 200 participants.

In determining effectiveness, the quality of a study should be considered due to risk of various forms of bias previously mentioned. While this research employed a simple grading system, some systematic reviews used more rigorous grading, such as the RE-AIM framework, [17, 33–35, 42] and others did not report a specific grading system [32, 39]. Random controlled trials are seen as best evidence in judging observed effects of an intervention on the predetermined outcome [46].

One final note, the fact that many different promotions run simultaneously adds a layer of difficulty in drawing conclusions as to the effectiveness of one intervention on outcomes measured [17, 32, 33, 35, 47]. An analysis of the effects of counseling on physical activity levels was undertaken but, two other physical activity interventions were simultaneously employed; multiple component programs have been shown to increase success [32, 47]. Thus, drawing a conclusion on the single effect of counseling is biased due to concurrent interventions.

### Attrition

Average attrition rates varied between 25 and 31% in the systematic reviews and individual studies, respectively. Investigating loss to follow-up is important as it can help determine the effectiveness and sustainability of a program as well as barriers to the uptake of interventions. Some of these studies noted reasons for dropouts [23, 48] while others gave no mention but recognized high attrition as a study design fault [8, 17, 30, 35]. In addition, dropout rates have been reported as typically higher among those that benefit the most from physical activity interventions. In general, dropouts are found to have a higher BMI and/or report poorer health at baseline including higher fat mass and lower reported PA [30, 38, 47, 49]. Workplace physical activity programs targeting this particular group of at risk employees should be at the forefront of a well-designed intervention for permanent behavior change.

### Sustainability

Program sustainability is a primary aim in order to convert research into practice so policies can be justified. Often, interventions are put in place for a short period. Measurement practices and tools should be relevant to the working population giving researchers reliable data. Measurements are taken several times throughout the intervention (minimum at the beginning and end) and then, on occasion, several months post intervention. Adequate assessment periods with long-term follow-up of 12 months or more post intervention are strongly suggested. Only 3 of the individual studies and 4 of the systematic reviews reported a 12-month or longer follow-up. It appears that in the short-term, physical activity levels raise among those participating. Long-term adoption of the increased activity level should be the primary goal of all stakeholders. This sentiment was an expressed concern by many other reviews [17, 32, 35, 39, 42] but not a particular aim built into programs. There remains a lack of studies and promotions seeking to improve the sustainability of health behaviors. In order to make an impact on worker’s health, policies prepared with the goal of permanent adoption of programs should be a priority.

### Strengths and limitations

This is the first scoping review to specifically address outcomes that are measured in Workplace Physical Activity Promotions. Secondly, we assessed how outcomes were evaluated with the particular tools used in the studies making note of both subjective and objective forms. To our knowledge, this is the first time research such as this has been completed. A third strength lies in the fact that both individual studies as well as systematic reviews were considered in order to be more inclusive. Nevertheless, several limitations to this scoping review are noted. (1) Dates are limited to studies published after 2008. If the dates were broadened, more studies could have been included. This would have enabled changes and trends in these interventions to be seen. (2) Four main databases were searched for eligible individual studies and systematic reviews. Broadening the search to more databases could have yielded more studies to include in the scoping review. (3) There is a possibility of publication bias due to preference for positive results. Many publications prefer to publish studies with positive results rather than report on studies that lacked significant findings. (4) There is a lack of applicability with studies before 2008. Studies done before the Global Action Plan for Worker’s Health could yield different outcomes and evaluation tools. (5) It is difficult to assess interventions in systematic reviews. Reviews report results based on their particular criteria making information extraction for this scoping review difficult. (6) The grading method may not be in depth enough to consider all types of quality in studies. Scoping reviews do not consider bias as do systematic reviews therefore, this was not taken into account during the quality assessment. A more in depth system using study design, population size, blinding and use of a matched control group could be constructed.

## Conclusion

Current evidence remains mixed as to the effectiveness of workplace health promotions [17, 32–36, 39, 42]. The workplace appears to be a prime pathway to reach many sedentary adults but, lack of homogeneous, consistent, quality data exists to conclude if interventions are effective and sustainable. In this review, we analyzed studies conducted in the workplace that explicitly included a physical activity component. Of particular interest was identifying the outcomes measured as physical activity and how those outcomes were evaluated. Inconclusive evidence combined with multiple measured outcomes and the lack of standardized evaluation tools makes determining impacts difficult. Large heterogeneity exists in regard to data collection methods for the measured outcomes in most studies. Improvements to methodology can aid in the evaluation of WHPs thereby increasing the likelihood of employers finding such interventions effective for their staff. Consistent study designs with standard, validated evaluation tools will help to combat these issues. In this research, every study relied upon subjective questionnaires to gather information but, the heterogeneous nature led to unclear outcomes and comparison difficulties. The questionable nature of validity and reliability of the effects is noted. Standardizing questionnaires for particular outcomes could alleviate this concern as well as ensure studies and interventions have comparable data. The last several decades has seen an explosion of WHPs. As they gain momentum in the public health sector, development of a best practices guide is warranted in order to establish comparable, reliable and valid data collection across multiple workplace sizes, programs and geographical regions. Physical activity interventions based on research should ensure the involvement and ongoing support of all stakeholders. These improvements will ensure better tracking methods to justify programs allowing resources to be appropriately targeted leading to the best, most sustainable health outcomes.

## Additional files

Additional file 1: Electronic, Biologic, Self-report Evaluation tools and Outcome Measure. (DOCX 22 kb)

Additional file 2: Individual study Physical Activity Outcomes. (DOCX 18 kb)

Additional file 3: Review Physical Activity Outcomes. (DOCX 19 kb)

## Abbreviations

BF%: Body fat percentage
BMI: Body mass index
CBT: Cognitive behavior therapy
CDC: Centers for Disease Control
IPAQ(-LF): International Physical Activity Questionnaire (−long form)
METs: Metabolic equivalents
MVPA: Moderate to vigorous physical activity
NCDs: Non-communicable diseases
NIH: National Institute of Health
OECD: Organization for Economic Co-operation and Development
PA: Physical activity
QoL: Quality of life
RCT: Randomized control trial
ROI: Return on investment
VO_2_ max: Maximal oxygen consumption
WHO: World Health Organization
WHP: Worksite health promotions
