# Supplementary material for: Understanding how outcomes are measured in workplace physical activity interventions: a scoping review
Source: BMC Public Health. 2018 Aug 25;18:1064. doi: 10.1186/s12889-018-5980-x (PMC6109358; doi:10.1186/s12889-018-5980-x)
Supplement: Supplementary file 1 [file 12889_2018_5980_MOESM1_ESM.docx]

## OUTCOME MEASURES/EVALUATION TOOLS

| | Physical activity behaviour/ level | Fitness | Motivation/Involvement/Self-efficacy | Nutrition | Anthropometric | Bio-Chemical Parameters | Work Related | Management | Health Related Quality of Life |
|---|---|---|---|---|---|---|---|---|---|
| **Electronic Tools** | Ergometer (2)<br>Accelerometer (6)<br>Activity Tracker (3)<br>Pedometer (12)<br>Inclinometer (2)<br>Cycling GPS (1) | | | | BMI (15)<br>Weight (7)<br>Body Fat % (9)<br>Circumference (6) | | | | |
| **Biologic Tools** | | VO2max (3)<br>Vmax (1)<br>Muscle Strength/Power (1)<br>Flexibility (1)<br>Endurance (1)<br>Cardiorepiratory Test (2)<br>Mobility/Balance (1) | | | | Heart Rate (3)<br>Blood Pressure (6)<br>Cholesterol (4)<br>Blood Glucose (1)<br>Lipids/Triglycerides (3)<br>Blood Plasma (1)<br>Bone Mineral (1) | | | |
| **Declarative Tools** | Diary (11)<br>IPAQ (6)<br>METs (6)<br>Modified Surveys (4)<br>Stages of change (2)<br>Stanford Exercise Behaviour (2)<br>Energy Performance Scan (1)<br>Efforts to Increase PAQ (1)<br>7-Day Recall Questionnaire (3)<br>AQ for Adults & Adolescents (1)<br>Workforce Sitting Questionnaire (1)<br>Godin Leisure Time PAQ (2)<br>PA at Work (1)<br>SQUASH (1)<br>PAQ for frequency/duration of physical activity (1)<br>Exercise behavior checklist (1)<br>Study Specific Questionnaires (1)<br>Undisclosed Questionnaires (2)<br>Paffenbarger PAQ (1)<br>Interviews (1) | | 28-item motivation to participate<br>Sport motivation scale (1)<br>Motivation for PA measure; rev(1)<br>Barriers to participate (2)<br>Adapted Likert scale (2)<br>Exercise Self-efficacy Scale (1)<br>Exercise Benefit/Barrier Scale (1)<br>Basic Psychological Needs in Exercise BPNE (1)<br>Behavioural Regulation in Exe (2)<br>Motivation to engage (2) | Nutritional Knowledge Survey (1)<br>Energy Performance Scan (1)<br>Dietary Behavior Survey (1)<br>Lifestyle Habitual Food Intake (1)<br>Stages of Change (1) | | | Absenteeism HR (6)<br>Energy Performance Scan (1)<br>Absenteeism Self report (1)<br>Working Relations Survey(1)<br>Health Care Use records (1)<br>Job Satisfaction Survey (1)<br>Job Stress Survey (1)<br>Work Limitations Questionnaire short form (1)<br>Work Performance (2)<br>Work ability (2)<br>Vitality (1)<br>Work Engagement (2)<br>Work Recovery (1)<br>Health and work Questionnaire (1)<br>Worksite injury report (1) | Management Vitality Perception Scan (1)<br>Heart Check adapted scale (1) | Quality of Life- Perceived Health (3)<br>Quality of Life Mood (1)<br>Health Survey 12-SF (3)<br>Adverse events (2)<br>Perceived Well-Being (1)<br>Quality of Life(SF-36) (2)<br>Musculoskeletal Pain (3)<br>EQ-5D (2)<br>Satisfaction with life scale (1)<br>Social support inventory (1)<br>Percieved Sleep (1)<br>Rosenberg Self-Esteem Scale (1)<br>Health Risk Assessment (1)<br>The multi-dimensional self-esteem inventory (1)<br>Epworth Sleepiness Scale(1)<br>Pittsburg Sleep Quality Index (1)<br>Energy Performance Scan (1) |
| | **26** | **7** | **9** | **5** | **4** | **7** | **15** | **2** | **17** |

*Numbers in () represent the number of studies utilizing tool

**Note each systematic review is only counted as once using tool regardless of number of studies in each review
