# Supplementary material for: Understanding how outcomes are measured in workplace physical activity interventions: a scoping review
Source: BMC Public Health. 2018 Aug 25;18:1064. doi: 10.1186/s12889-018-5980-x (PMC6109358; doi:10.1186/s12889-018-5980-x)
Supplement: Supplementary file 2 [file 12889_2018_5980_MOESM2_ESM.docx]

Study	Physical Activity	Evaluation Tool(s)	Objective or Subjective Measure	Validation	Unit of Measure
**Individual Studies**					
Davey, J., et al.	10,000 Steps	Pedometer<br>Modified Active for Life/Healthy People survey	Objective<br>Subjective	Validated<br>Not validated	Steps
Dishman, R. , et al.	MVPA	Pedometer<br>IPAQ	Objective<br>Subjective	Validated<br>Validated	Steps<br>METs
Maruyama, C., et al.	Increase Steps	Pedometer<br>Stages of Change Questionnaire<br>Efforts to Increase Physical Activity Questionnaire	Objective<br>Subjective<br>Subjective	Validated<br>Unknown<br>Not validated	Steps
McEachan, R., et al.	MVPA	IPAQ-SF	Subjective	Validated	METs
Morgan, PJ, et al.	Weight Change	Pedometer<br>Online Exercise diary	Objective<br>Subjective	Validated<br>Not validated	Steps<br>METs
Edries, N., et al.	Structured Exercise	Stanford Exercise Behavior Scale	Subjective	Validated	Minutes of exercise
Chae, D., et al.	Changes in Physical Activity	Pedometer	Objective	Validated	Steps
Brakenridge, C.L., et al.	Reduce sitting time	Activity Tracker<br>Online Exercise Diary	Objective<br>Subjective	Validated<br>Not validated	Steps<br>Sitting time
Hendricksen, I., et al.	MVPA	Energy Performance Scan Questionnaire	Objective	Validated	Minutes in MVPA/wk<br>Sedentary min/workday
Hori, H., et al.	10,000 Steps	Pedometer	Objective	Validated	Steps
Aittasalo, M., et al.	Leisure PA<br>Reduce sitting time	Accelerometer<br>Modified Finnish Population Survey<br>Workforce Sitting Questionnaire<br>Diary	Objective<br>Subjective<br>Subjective<br>Subjective	Validated<br>Unknown<br>Validated<br>Not Validated	Minutes in MVPA/wk<br>Sedentary min/workday
Arrogi, A., et al.	MVPA<br>Reduce sitting time	Activity Tracker<br>Pedometer<br>IPAQ-SF	Objective<br>Objective<br>Subjective	Validated<br>Validated<br>Validated	Steps<br>METs

*Unknown: validation is questionable in this population
