# Supplementary material for: Understanding how outcomes are measured in workplace physical activity interventions: a scoping review
Source: BMC Public Health. 2018 Aug 25;18:1064. doi: 10.1186/s12889-018-5980-x (PMC6109358; doi:10.1186/s12889-018-5980-x)
Supplement: Supplementary file 3 [file 12889_2018_5980_MOESM3_ESM.docx]

Study	Physical Activity	Evaluation Tool(s)	Objective or Subjective Measure	Validation	Unit of Measure
**Reviews**					
Conn, V., et al.	Steps<br>Minutes of Exercise	Ergometer<br>Diary	Objective<br>Subjective	Validated<br>Not validated	Steps<br>Min/#/frequency of supervised & unsupervised exercise
Vuillemin, A., et al.	Increased Physical Activity<br>Increased Physical Fitness	Accelerometer<br>Pedometer<br>Cycling GPS<br>Ergometer<br>IPAQ<br>Godin Leisure Time PAQ<br>Physical Activity at Work Questionnaire<br>Modified MONICA Questionnarie<br>7-Day Recall Questionnaire<br>Diary<br>Stages of Change	Objective<br>Objective<br>Objective<br>Objective<br>Subjective<br>Subjective<br>Subjective<br>Subjective<br>Subjective<br>Subjective<br>Subjective	Validated<br>Validated<br>Validated<br>Validated<br>Validated<br>Validated<br>Validated<br>Unknown<br>Validated<br>Not validated<br>Validated	Steps<br>Cycling distance/time<br>Sit time minutes<br>% stair use<br>Muscle strength<br>Muscle endurance<br>Flexibility<br>VO2max
Schroer, S., et al.	Steps<br>Minutes of Exercise<br>Active Commuting<br>Sports	Pedometer<br>Diary	Objective<br>Subjective	Validated<br>Not validated	Steps<br>METs<br>VO2max<br>% stair use
Amlani, N., et al.	Steps<br>MVPA<br>Minutes of Exercise<br>Sports	Accelerometer<br>Diary	Objective<br>Subjective	Validated<br>Not validated	Steps<br>METs
Malik, S., et al.	Walking<br>MVPA	Accelerometer<br>Pedometer<br>Inclinometer<br>IPAQ<br>7-Day Recall<br>Diary<br>Study specific designed questionnaire	Objective<br>Objective<br>Objective<br>Subjective<br>Subjective<br>Subjective<br>Subjective	Validated<br>Validated<br>Validated<br>Validated<br>Not validated<br>Not validated<br>Not validated	Steps<br>METs
Brinkley, A., et al.	Steps<br>Minutes of Exercise<br>Sports participation	Pedometer<br>Undisclosed online survey<br>Undisclosed Questionnaires<br>Interviews<br>Diary	Objective<br>Subjective<br>Subjective<br>Subjective<br>Subjective	Validated<br>Unknown<br>Unknown<br>Not validated<br>Not validated	Steps<br>METs<br>VO2max<br>Perceived fitness/activity level increase
Shrestha, N., et al.	Steps<br>Reduced sitting time	Accelerometer<br>Pedometer<br>Inclinometer<br>Diary	Objective<br>Objective<br>Objective<br>Subjective	Validated<br>Validated<br>Validated<br>Unknown	Steps<br>Sit time minutes
Reed, J.L., et al.	Steps<br>MVPA	Accelerometer<br>Activity Tracker<br>IPAQ<br>SQUASH<br>Godin leisure time exercise questionnaire<br>Standford PA recall<br>PAQ for frequency/duration of PA<br>Paffenbarger PAQ<br>7-day recall<br>Activity questionnaire for adults & adolescents<br>Exercise behavior checklist<br>Diary<br>Adapted cross-cultural activity patterns questionnaire	Objective<br>Objective<br>Subjective<br>Subjective<br>Subjective<br>Subjective<br>Subjective<br>Subjective<br>Subjective<br>Subjective<br>Subjective<br>Subjective<br>Subjective	Validated<br>Validated<br>Validated<br>Unknown<br>Validated<br>Validated<br>Unknown<br>Validated<br>Validated<br>Unknown<br>Unknown<br>Not validated<br>Unknown	Steps<br>METs<br>Minutes in MVPA/wk<br>Activity Score

*Unknown- validation is questionable in this particular population
